# Effect of Long-Chain Polyunsaturated Fatty Acid Supplementation on Neurodevelopmental Outcome in Full-Term Infants 

**DOI:** 10.3390/nu2080790

**Published:** 2010-07-27

**Authors:** Mijna Hadders-Algra

**Affiliations:** Department of Pediatrics–Developmental Neurology, University Medical Center Groningen, Hanzeplein 1, 9713 GZ Groningen, The Netherlands; Email: m.hadders-algra@med.umcg.nl; Tel.:+31 50 3614247; Fax: +31 50 3619158.

**Keywords:** LCPUFA, docosahexaenoic acid, arachidonic acid, full-term, infant, neurodevelopment, cognition, breast feeding

## Abstract

It takes more than 20 years before the human brain obtains its complex, adult configuration. Most dramatic developmental changes occur prenatally and early postnatally. During development, long-chain polyunsaturated fatty acids (LCPUFA) such as doxosahexaenoic acid (DHA) and arachidonic acid (AA) are accreted in the brain. Since breastfeeding is associated with a better developmental outcome than formula feeding, and human milk in contrast to traditional standard formula contains LCPUFA, the question arose whether LCPUFA supplementation of infant formula may promote the neurodevelopmental outcome. The current paper reviews the evidence available in full-term infants. It concludes that postnatal supplementation of formula with LCPUFA is associated with a beneficial effect on short-term neurodevelopmental outcome. However, no evidence is available that LCPUFA supplementation enhances neurodevelopmental outcome in full-term infants beyond the age of four months. Nevertheless, it should be realized that very limited information is available on the effect of LCPUFA supplementation on neurodevelopmental outcome at school age or later. It is conceivable that effects of LCPUFA supplementation first emerge or re-emerge at school age when more complex neural functions are expressed.

## 1. Introduction: Breastfeeding is Associated with Better Developmental Outcome

More than 80 years ago, it had already been noted that breastfed infants had a better developmental outcome than infants fed other types of milk [1]. Now it is well established that breastfeeding is associated with a better neurological, cognitive and behavioral outcome than formula feeding [2,3,4,5]. However, it has not been clarified to which extent the composition of human milk explains this difference in development. For example, when perinatal and social confounders are taken into account, breastfeeding is associated with an increment of cognitive function of about 3 IQ points [4]. However, when the results are also adjusted for maternal IQ the cognitive benefit of breastfeeding is reduced to a small and non-significant difference of about 0.5 IQ points [6]. On the other hand, breastfeeding remained associated with a lower prevalence of fine manipulative dysfunction when maternal IQ was taken into account [3]. In addition, the finding that prolongation of the duration of breastfeeding – as a result of a cluster-randomized trial to promote breastfeeding – was associated with a better cognitive outcome at six years [7], suggests that the composition of human milk plays a role in the positive association between breastfeeding and outcome. Until recently, one of the differences between human milk and standard commercially available formulas was the presence of long-chain polyunsaturated fatty acids (LCPUFA) in human milk [8]. This fact inspired research on the effect of LCPUFA supplementation of formula in pre-term and full-term infants. The present paper aims at reviewing the effect of LCPUFA supplementation on neurodevelopmental outcome in full-term infants. The paragraphs reviewing the studies on the effect of LCPUFA supplementation are preceded by sections summarizing the ontogeny of the human brain and fatty acid accretion in the central nervous system.

## 2. Ontogeny of the Human Brain

The development of the human brain takes over 20 years. It is based on a continuous interaction between genetic information and environmental factors (for details on human brain ontogeny see [9,10,11]). Genetic instructions are, for instance, the major driving forces behind the functional topography of the human brain [12]. Environmental influences may vary from external sensory experiences, sensory experiences generated by self-produced motor activity and effects of chemical substances. The latter may be generated endogenously by the body or obtained from the external world, such as nutritional substances.

Brain development starts during the early phases of gestation with the proliferation of neurons in the germinal layers near the ventricles [13]. Next, neurons migrate in an orderly fashion to their final destinations. Meanwhile, they start to differentiate [14]. Neuronal differentiation includes the formation of dendrites and axons, the production of neurotransmitters and synapses, and the elaboration of the intracellular signaling machinery and the complex neural membranes [9,10]. The process of differentiation is particularly active in the few months prior to term age and the first few months post-term. The formation of synapses continues throughout life. Besides neural cells, glial cells are generated. The peak of glial cell production occurs in the second half of gestation [15]. Part of the glial cells take care of axonal myelination. Myelination takes place especially between the second trimester of gestation and the end of the first postnatal year. But it is first completed around the age of 40 years [16]. A remarkable feature of brain development is that it does not only consist of creation of components, but also of an elimination of elements. About half of the created neurons die off (apoptosis), in particular during mid-gestation [17]. Similarly, axons and synapses are eliminated, the latter especially between the onset of puberty and early adult age [18,19]. The shaping of the nervous system by these regressive phenomena is guided by neurochemical processes and neural activity [20]. The neural elements which fit the environment best persist, thus allowing for an adaptation of the brain to its own environment. 

Brain development is characterized by phases of transition. A major transition occurs around three to four months post-term [21,22,23]. At three to four months, the subplate, the transient cortical structure mediating fetal and neonatal behavior, has been largely replaced by the cortical plate [22]. The cortical changes are paralleled by changes in behavior [21]. For instance, motor behavior changes from motility dominated by non-goal directed general movements into goal directed motility such as reaching behavior [24]. 

The continuous developmental changes in the brain have three practical implications for studies on the effect of LCPUFA supplementation. First, the effects of supplementation will depend on the timing of the supplementation. In other words, the effect of postnatal supplementation in pre-terms may differ from that in full-term infants. Second, the neurodevelopmental instruments to assess the effect of intervention should be age-specific. Third, the effects of supplementation on neurodevelopmental outcome in early infancy may differ from those on outcome at later age. Highly relevant for studies on the effect of nutritional intervention during early life is the finding that the development of neural dysfunction due to prenatal, perinatal or neonatal adversities may be characterized by phases in which dysfunction is not expressed. For instance, the Groningen Perinatal Project demonstrated that prenatal, perinatal and neonatal adversities were strongly associated with neonatal neurological morbidity. However, at the age of 18 months, the association between early risk factors and neurological dysfunction had disappeared to a large extent. It was first at school age, in particular from nine years onwards, that clear associations between early adversities and minor neurological dysfunction, learning and behavioral problems re-emerged [25]. 

## 3. Fatty Acid Accretion in the Human Brain

Fatty acids are major components of brain tissue: about 50 to 60% of dry adult brain weight consists of fatty acids [26]. Long-chain polyunsaturated fatty acids (LCPUFA), such as doxosahexaenoic acid (DHA; 22:6ω3) and arachidonic acid (AA; 20:4ω6) are amongst the most important fatty acids which are incorporated into the brain, in particular into the synaptic membranes [26,27,28]. Initially LCPUFA accretion in human neural tissue proceeds rather slowly [29]. An LCPUFA accretion spurt first occurs in the last trimester of gestation [30,31]. There is some evidence that accretion of AA in the brain during the last trimester exceeds that of DHA, so that at term age the brain contains relatively more AA than DHA [30,31]. After term age, LCPUFA accretion in the nervous system continues. Gradually accretion of DHA surpasses that of AA. As a result, DHA is the major LCPUFA in the adult brain. In the brain LCPUFA accumulate mainly in the cortical gray matter, in particular in the synaptic membranes, and to a lesser extent in the white matter [27,28]. The baboon study of Diau *et al.* [27] indicated that highest concentrations of DHA and AA are found in the precentral, postcentral, prefrontal and occipital cortices, basal ganglia, hippocampus, thalamus and cerebellum, a finding which suggests that LCPUFA intake might affect in particular circuitries involved in sensorimotor integration, attention-executive function and memory.

DHA and AA can be obtained directly from the diet or by endogeneous conversion of the parent precursors alpha-linolenic acid (ALA; 18:3ω3) and linoleic acid (LA; 18:2ω6) by means of chain elongation and desaturation. The fetus and newborn infant are capable of these conversion processes, but the enzymatic systems involved seem to be unable to supply sufficient LCPUFA to meet the high requirements until 16 weeks after term age [28]. This means that during early development, LCPUFA supply is largely dependent on dietary intake of DHA and AA. For the fetus, this implies that it is dependent on maternal intake of LCPUFA, while the young infant has to rely on LCPUFA in milk [32,33]. 

## 4. LCPUFA Supplementation and Development in Full-Term Infants

The systematic review of Simmer *et al.* [34] concluded that the RCTs on the effect of postnatal LCPUFA supplementation of formula milk in term infants did not show a beneficial effect of LCUPFA on neurodevelopmental outcome. However, this conclusion may be slightly modified when age at outcome is taken into account. The effect of LCPUFA supplementation on outcome in early infancy differs from that after the age of four months. This is illustrated in Table 1, which summarizes outcome until four months, and Table 2, which addresses outcome after the age of four months. The studies are rank ordered according to the level of DHA supplementation. Most studies evaluated visual development, cognitive or motor development. Table 1 shows that one of the three studies, in which formulae containing less than 0.30% DHA were evaluated, reported a better outcome in the supplemented infants, whereas seven out of eight studies in which formula with ≥0.30% DHA were used demonstrated a better developmental outcome in supplemented infants. On the basis of the limited number of studies, in particular those which studied the effect of relatively low DHA dosages, it can not be determined whether a beneficial effect of DHA supplementation on developmental outcome at early age depends on DHA dosage. Nevertheless, the ‘meta-regression analysis’ of Uauy *et al.* [35] suggested that the effect of postnatal LCPUFA supplementation in term infants on early visual development may be dependent on DHA dosage. It is also conceivable that a dosage of about 0.30% DHA may be sufficient to achieve the effect, as the recent study of Birch *et al.* [37] indicated that the effect of DHA on visual development was similar for supplementation with 0.32%, 0.64% or 0.96% DHA. Table 2 indicates, however, that the positive effect of postnatal LCPUFA supplementation on developmental outcome could not be demonstrated for outcome after the age of four months.

**Table 1:** LCPUFA supplementation in term infants and outcome until four months of age (adapted and updated from Hadders-Algra 2005 [36]).

Author(s)	Groups	Duration of supplementation	DHA content	AA content	Age at FU (months)	Attrition at last FU	Assessment at follow-up	Results
Carlson *et al.* 1996 [37]	E n = 19	?	0.10%	0.43%	2 and 4	38%	Teller visual acuity [38]	2 mo: E > C; BF > C
	C n = 20							4 mo: E = C; BF = C
	BF n = 19	BF ≥ 3 mo						
Auestad *et al.* 1997 [39] ^a^	E_1_ n = 26	≥ 4 mo	0.12%	0.43%	2 and 4	39%	FPL [40]	E_1 _= C; E_2 _= C
	E_2_ n = 28		0.20%			(at 12 mo)	Sweep VEP [41]	BF = C
	C n = 28							
	BF n = 38	BF ≥ 4 mo						
Auestad *et al.* 2001 [42]	E_1_ n = 58	12 mo	0.14% (egg)	0.45%	1, 2 and 4	27%	Teller visual acuity [38]	E_1_ = C; E_2 _= C
	E_2_ n = 60		0.13% (fish/fungal)	0.46%		(at 12 mo)		BF = C
	C n = 56							
	BF n = 120	BF ≥ 3 mo						
Agostoni *et al.* 1995 [43]	E n = 27	4 mo	0.30%	0.44%	4	4%	Brunet –Lezine DQ [44]	E > C
	C n = 29							BF > C
	BF n = 30	BF ≥ 4 mo						
Bouwstra *et al.* 2003 [45]^b^	E n = 131	2 mo	0.30%	0.45%	3	16%	Quality of general movements [46]	E > C
	C n = 119	BF variable;						BF > C
	BF n = 147	median 9 wk						
Makrides *et al.* 2000 [47]	E_1_ n = 24	12 mo	0.34%	0.34%	4	18%	VEP [41]	E_1_ = C; E_2 _= C
	E_2_ n = 23		0.35%			(at 8 mo)		BF = C
	C n = 21							
	BF n = 46	BF ≥ 3 mo						
Makrides *et al.* 1995 [48]	E n = 13	?	0.36%		4	11%	VEP [41]	E > C
	C n = 19							BF > C
	BF n = 23	BF ≥ 4 mo						
Birch *et al.* 1998 [49]	E_1_ n = 23	4 mo	0.36%	0.72%	1.5 and 4	18%	FPL [40]	E = C; BF = C
	E_2_ n = 22		0.35%					
	C n = 23						Sweep VEP [41]	E_1_ > C; E_2_ > C
	BF n = 21	BF ≥ 4 mo						BF > C
Birch *et al.* 2005 [50]	E n = 46	12 mo	0.36%	0.72%	1.5 and 4	11%	Sweep VEP [41]	E > C
	C n = 46							
					4		Stereoacuity [51]	E > C
Ünay *et al.* 2004 [52]	E n = 22	4 mo	0.50%		4	16%	BAEP [53]	E > C
	C n = 22							BF > C
	BF n = 23	BF ≥ 4 mo						
Birch *et al.* 2010* [54]	E_1_ n = 74	12 mo	0.32%	0.64%	1.5 and 4	12%	Sweep VEP [41]	E_1_ = E_2_ = E_3_ > C
	E_2_ n = 75		0.64%					
	E_3_ n = 75		0.96%					
	C n = 76							

AA = arachidonic acid, BAEP = brainstem auditory evoked potential, BF = breast fed group, C = control group, DHA = docosahexaenoic acid, DQ = developmental quotient, E = experimental group, FPL = forced preference looking, FU = follow-up, mo = months, VEP = visual evoked potential. E = C: no statistically significant difference between experimental and control group; E > C: experimental group significantly better outcome than controls; E < C: experimental group significantly worse than control group. Superscript letters in authors column indicate that studies evaluated outcome in more or less the same study groups. * Birch *et al.* 2010: no exact numbers on assessed infants at each age available.

**Table 2:** LCPUFA supplementation in term infants and outcome beyond 4 months (adapted and updated from Hadders-Algra 2005 [26]).

Author(s)	Groups	Duration of supplementation	DHA content	AA content	Age at FU (months)	Attrition at last FU	Assessment at follow-up	Results
Carlson *et al.* 1996 [37]	E n = 19	?	0.10%	0.43%	6 and 12	≥ 38%	Teller visual acuity [38]	E = C
	C n = 20							BF = C
	BF n = 19	BF ≥ 3 mo						
Auestad *et al.* 1997 [39] ^a^	E_1_ n = 26	≥ 4 mo	0.12%	0.43%	6, 9 and 12	39%	FPL [40]	E = C
	E_2_ n = 28		0.20%				Sweep VEP [41]	BF = C
	C n = 28							
	BF n = 38	BF ≥ 4 mo						
Scott *et al.* 1998 [55] ^a^	E_1_ n = 38	≥ 4 mo	0.12%	0.43%	12	37%	Bayley PDI / MDI [56]	E_1_ = C; E_2_ = C; BF = C
	E_2_ n = 33		0.20%				McArthur language [57]	
	C n = 42				14			E_1_ = C; E_2_ < C; BF = C
	BF n = 60	BF ≥ 3 mo						
Auestad *et al.* 2001 [42]	E_1_ n = 58	12 mo	0.14% (egg)	0.45%	6, 9, 12	27%	Teller vis. acuity [38]	E = C; BF = C
	E_2_ n = 60		0.13% (fish/fungal)	0.46%		(at 12 mo)		
	C n = 56				6 and 9		Fagan [58]	E = C; BF = C
	BF n = 120	BF ≥ 3 mo						
					6 and 12		Bayley PDI / MDI [56]	E = C; BF = C
		
					6 and 12		IBQ [59]	E = C; BF = C
		
					9 and 14		Language [57]	E = C; BF = C
Willats *et al.* 1998 [60]	E n = 21	4 mo	0.20	0.30	10	38%	Problem solving task [60]	E > C
	C n = 23							
Auestad *et al.* 2003 [61] ^a^	E_1_ n = 35	12 mo	0.12%	0.43%	39	47%	Teller vis. acuity	E = C; BF = C
	E_2_ n = 35		0.23%				Beery VMI [62]	E = C; BF = C
	C n = 37						Stanford- Binet IQ [63]	E = C; BF = C
	BF n = 50	BF ≥ 3 mo					Language [57]	
								E = C; BF = C
Bouwstra *et al.* 2005 [64]^ b^	E n = 135	2 mo	0.30%	0.45%	18	6%	Hempel neurolog exam [65]	E = C; BF = C
	C n = 157	BF variable; median 9 wk						
	BF n = 154						Bayley PDI / MDI [56]	
De Jong *et al.* 2010 [3]^ b^	E n = 91	2 mo	0.30%	0.45%	9 yr	28%	Touwen neurological exam [66]	E = C; BF > C
	C n = 123	BF variable; median 9 wk						
	BF n = 127							
Agostoni *et al.* 1997 [67]	E n = 26	4 mo	0.30%	0.44%	24	10%	Brunet – Lezine DQ [44]	E = C; BF = C
	C n = 30							
	BF n = 25	BF ≥ 4 mo						
Lucas *et al.* 1999 [68]	E n = 125	6 mo	0.32%	0.30%	9	21%	Knobloch DQ [69]	E = C; BF = C
	C n = 125						Bayley PDI / MDI [56]	
	BF n = 104	BF ≥ 6 wk			18			E = C; BF = C
Singhal *et al.* 2007 [70]	E n = 89	6 mo	0.32%	0.30%	4-6 yr	46%	Visual acuity [71]	E = C; BF = C
	C n = 81							
	BF n = 73	BF ≥ 6 wk			4-6 yr		Stereoacuity [51]	E = C; BF > C
Makrides *et al.* 2000 [47]	E_1_ n = 19	12 mo	0.34%	0.34%	8	12%	VEP [41]	E = C; BF = C
	E_2_ n = 22		0.35%					
	C n = 19				12 and 24		Bayley PDI / MDI [56]	PDI: E = C; BF = C
	BF n = 23	BF ≥ 3 mo						12 mo MDI: E = C; BF = C; 24 mo MDI: E = C; BF > C
Birch *et al.* 1998 [49]	E_1_ n = 19	4 mo	0.36%	0.72%	6 and 12	26%	FPL [40]	E = C; BF = C
	E_2_ n = 22		0.35%				Sweep VEP [41]	6 mo VEP: E = C;
	C n = 20							BF = C; 12 mo VEP:
	BF n = 46	BF ≥ 4 mo						E_1 _> C; E_2 _> C; BF >C
Birch *et al.* 2000 [72]	E_1_ n = 19	4 mo	0.36%	0.72%	18	29%	Bayley PDI / MDI [56]	MDI: E_1 _> C, E_2 _= C
	E_2_ n = 17		0.35%					PDI: E = C
	C n = 20							
Birch *et al.* 2007 [50]	E_1_ n = 19	4 mo	0.36%	0.72%	4 yr	34%	visual acuity [74]	E_1_= C, E_2 _> C, BF > C
	E_2_ n = 17		0.35%				WPPSI [75]	VIQ: E = C, BF > C, E_1_
	C n = 20							PIQ: E = C, BF =C
	BF n = 32							
Birch *et al.* 2005 [35]	E n = 42	12 mo	0.36%	0.72%	9 and 12	17%	Sweep VEP [41]	E > C
	C n = 44							
					9 and 12		Stereoacuity [51]	E = C
Birch *et al.* 2010* [54]	E_1_ n = 64	12 mo	0.32%	0.64%	9 and 12	28%	Sweep VEP [41]	E_1_ = E_2 _= E_3 _> C
	E_2_ n = 59		0.64%					
	E_3_ n = 65		0.96%					
	C n = 56							

Note that some of the studies of Table 2 are also included in Table 1; this implies that the groups were re-assessed after the age of four months. AA = arachidonic acid, BF = breast fed group, C = control group, DHA = docosahexaenoic acid, DQ = developmental quotient, E = experimental group, FPL = forced preference looking, FU = follow-up, IBQ = infant behavior questionnaire, IQ = intelligence quotient, MDI = mental developmental index, mo = months, PDI = psychomotor developmental index, VEP = visual evoked potential, VMI = visuomotor integration, WPPSI = Wechsler Preschool and Primary Scale of Intelligence E = C: no statistically significant difference between experimental and control group; E > C: experimental group significantly better outcome than controls; E < C: experimental group significantly worse than control group. Superscript letters in authors column indicate that studies evaluated outcome in more or less the same study groups. * Birch *et al.* 2010: no exact numbers on assessed infants at each age available.

## 5. Concluding Remarks

Postnatal supplementation of formula with LCPUFA in full-term infants is associated with a beneficial effect on short term neurodevelopmental outcome. Possibly, this short term beneficial effect is brought about by DHA levels of at least 0.30%. Interestingly, this putative threshold level is similar to the average level of DHA in human milk worldwide (0.32%, [8]). No long-term beneficial effects of LCPUFA supplementation in full-term infants have been demonstrated. However, it should be realized that very limited information is available on the effect of LCPUFA supplementation on neurodevelopmental outcome at school age or later. It is conceivable that effects of LCPUFA supplementation first emerge or re-emerge at school age when more complex neural functions are expressed. The latter possibility may be illustrated by the age-dependency of developmental differences between breastfed and formula-fed infants in the Netherlands. At the age of three months breast-fed infants have a better neurological condition than formula-fed infants [45], at 18 months this difference has disappeared [64], to re-emerge at the age of at least 3½ years [3,76] 
